# A Unique Case of Conservatively Treated Actinomyces Empyema Complicated Due to Drug Reaction With Eosinophilia and Systemic Symptoms Syndrome

**DOI:** 10.7759/cureus.41954

**Published:** 2023-07-16

**Authors:** Ilias E Dimeas, Sotirios Sinis, George Dimeas, Stratos Skrimizeas, Zoe Daniil

**Affiliations:** 1 Respiratory Medicine, University Hospital of Larissa, Larissa, GRC

**Keywords:** drug-induced eosinophilia, bilateral pleural effusion, pulmonary actinomycosis, pulmonary empyema, drug reaction with eosinophilia and systemic symptoms (dress) syndrome, actinomyces infection

## Abstract

This case contemplates the unusual presentation, challenging diagnostic workup and conservative therapeutic process of a patient with Actinomyces empyema complicated along the way due to drug reaction with eosinophilia and systemic symptoms (DRESS) syndrome. The patient was a 40-year-old male, who presented with pleurodynia and fever. Laboratory exams showed elevated inflammatory markers and imaging revealed two biconvex fluid pockets located in the right lower lobe, from which the fluid was positive for Actinomyces meyeri. The initial conservative process with intravenous antibiotics and successful drainage with intrapleural fibrinolysis improved our patient. However, after a few days, the patient’s fevers relapsed, and as regress of the empyema was discussed as a complication, he developed a maculopapular symmetrical rash of the trunk and legs accompanied by enlarged lymph nodes, eosinophilia, thrombocytopenia, and atypical lymphocytes. The diagnosis of DRESS syndrome due to antibiotic therapy for actinomyces empyema was established and a balance between bactericidal and immunosuppression medication had to be found. Fortunately, the patient withstood prolonged antibiotic therapy and got fully treated without any relapses.

## Introduction

Pulmonary actinomycosis frequently develops in middle-aged males, especially those with a history of alcohol abuse and structural lung diseases. The clinical appearance is nonspecific and includes fever and chest pain [1]. Diagnosis of this condition is elusive often leading to delayed treatment which may culminate in pernicious sequelae such as empyema necessitans. Minimally invasive techniques, such as thoracic tube placement instead of pleura decortication, along with long-term antibiotic therapy, for at least three months, might be the ideal scenario when there is an early diagnosis of actinomyces empyema [2]. Even then, sometimes severe cutaneous adverse reactions such as drug reaction with eosinophilia and systemic symptoms (DRESS) syndrome complicate the treatment plan as immunosuppression might be mandatory to avoid a potentially life-threatening inflammation of internal organs.

## Case presentation

A 40-year-old shepherd (smoker, 35 pack/years) was referred to the emergency department because of right hemithorax pleurodynia and fever. At pleurodynia onset one month prior, the patient was prescribed analgesics and muscle relaxants for symptomatic relief. However, five days before his visit to the emergency department the patient became febrile and completed a course of moxifloxacin. No remarkable medical history was obtained except for daily consumption of alcohol and a tooth abscess that resolved spontaneously two months ago. The patient was admitted to the Department of Respiratory Medicine to be diagnosed and treated.

On admission, the patient was febrile (temperature 39.4oC). Peripheral lymph nodes were normal. During lung auscultation, right middle-lung-area coarse crackles were recognized while the right lower lung field was silent, devoid of any breath sounds with dullness upon percussion. No pathological heart sounds were detected. Oropharynx inspection revealed gingivitis and dental caries. No other abnormalities were found. 

The patient had hypoxemic respiratory failure [pO2=58mmHg (> 80mmHg), pCO2=36mmHg (35-45 mmHg), FiO2=21%] according to arterial blood gas analysis. Remarkable laboratory findings involved leukocytosis (28,2 x 103 cells/μL), anemia (hemoglobin concentration of 9,2 g/dL), reactive thrombocytosis (780 x 103 platelets/μL), elevated c-reactive protein (34 mg/dL, <0,05 mg/dL) and increased erythrocyte sedimentation rate (104 mm/hr).

**Figure 1:**
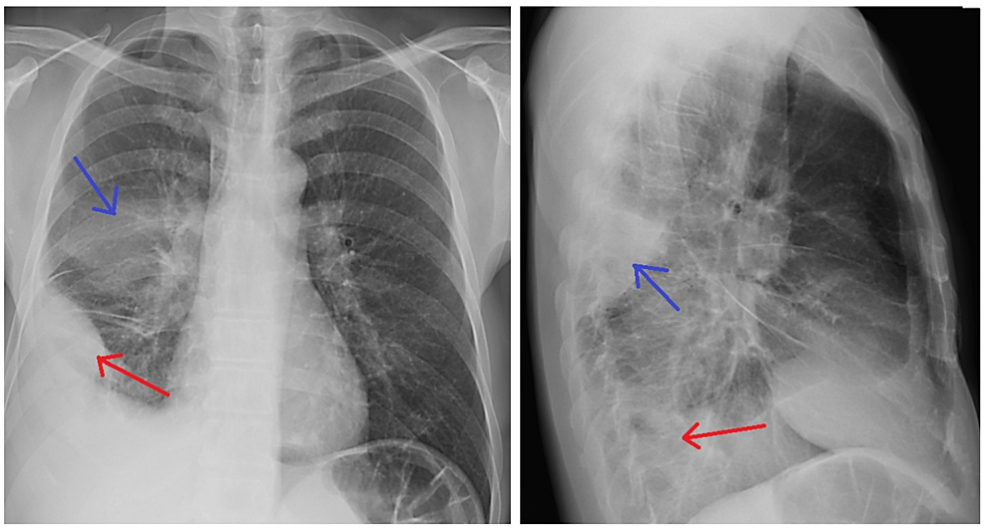
Admission chest x-ray Biconvex blunting of the right costodigraphmatic recess (red arrow) and triangular opacification in the right middle lung field by the visceral pleura (blue arrow).

**Figure 2:**
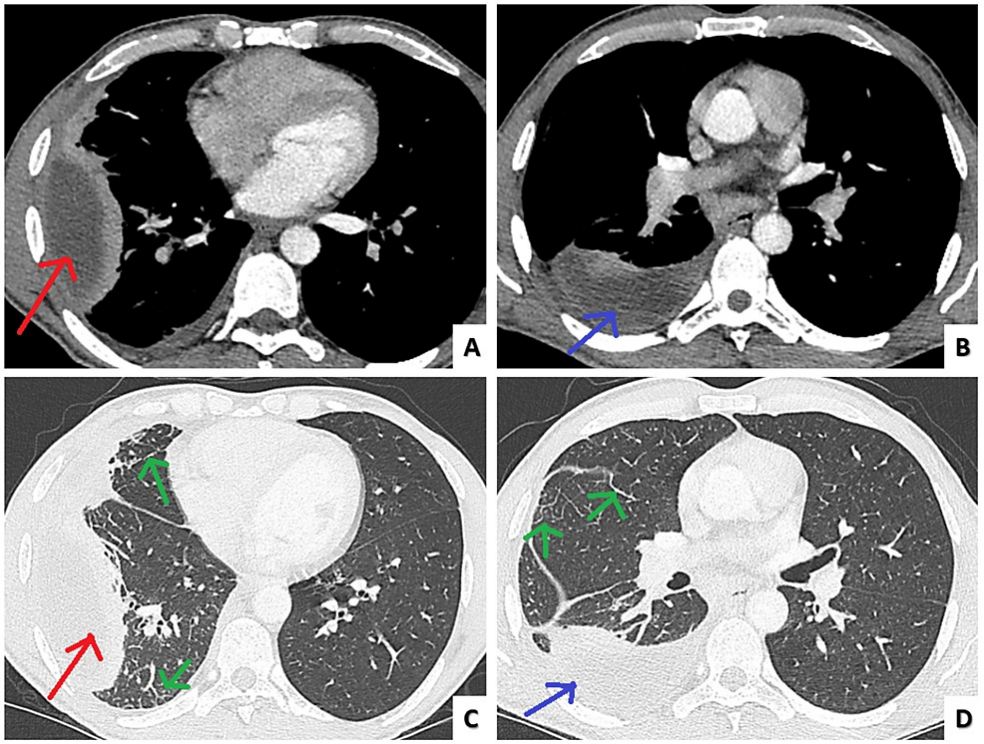
Admission chest computed tomography Two pockets of pleural effusion. The bigger one (9,6cm x 3,6cm x 5cm-red arrow) (A) located in the anterior and lateral segments of the right lower lobe and the smaller one (7,45cm x 2,85cm x 6,5cm-blue arrow) (B) in the superior segment of the right lower lobe. Mild interlobular septa thickening is noted (green arrows) in the right lung (C, D).

Chest radiography revealed a blunting of the right costodiagraphmatic recess and a triangular opacification in the right middle lung field by the visceral pleura (Figure 1). Chest computed tomography (Figure 2) showed two biconvex fluid pockets with positive split pleura sign [3], the larger one located in the anterior and lateral segments (pocket 1) of the right lower lobe and the smaller one in the superior segment of the right lower lob (pocket 2). A diagnostic thoracocentesis was performed and pleural fluid was purulent and putrid with a green tint.

As for further diagnostic studies, transthoracic echocardiography did not show evidence of infective endocarditis, blood and sputum cultures were negative, however microbiological and molecular studies of the pleural fluid were positive for Actinomyces meyeri. 

**Figure 3:**
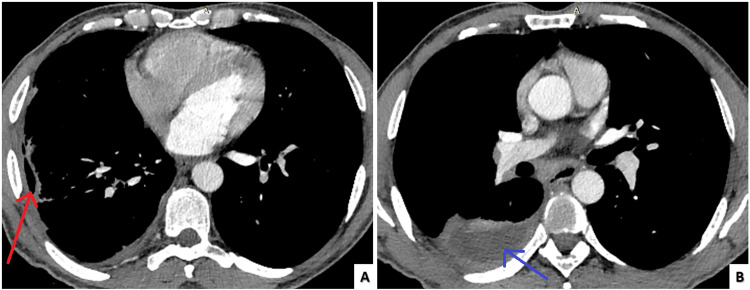
Inpatient follow-up chest computed tomography Full remission of the pocket (A) in the anterolateral segment of the right lower lobe (red arrow) and reduction of the pocket (B) in the superior segment of the right lower lobe (blue arrow).

The chest tube within pocket 1 was removed following successful drainage after three courses of intrapleural fibrinolysis which resulted in defervescence & improved gas exchange. After three weeks of intravenous piperacillin-tazobactam for extended anaerobic coverage beyond actinomyces, however, the fever relapsed. A neck/chest computed tomography was negative for cervicofacial or mediastinal actinomycosis and showed signs of empyema resolution (Figure 3).

**Figure 4:**
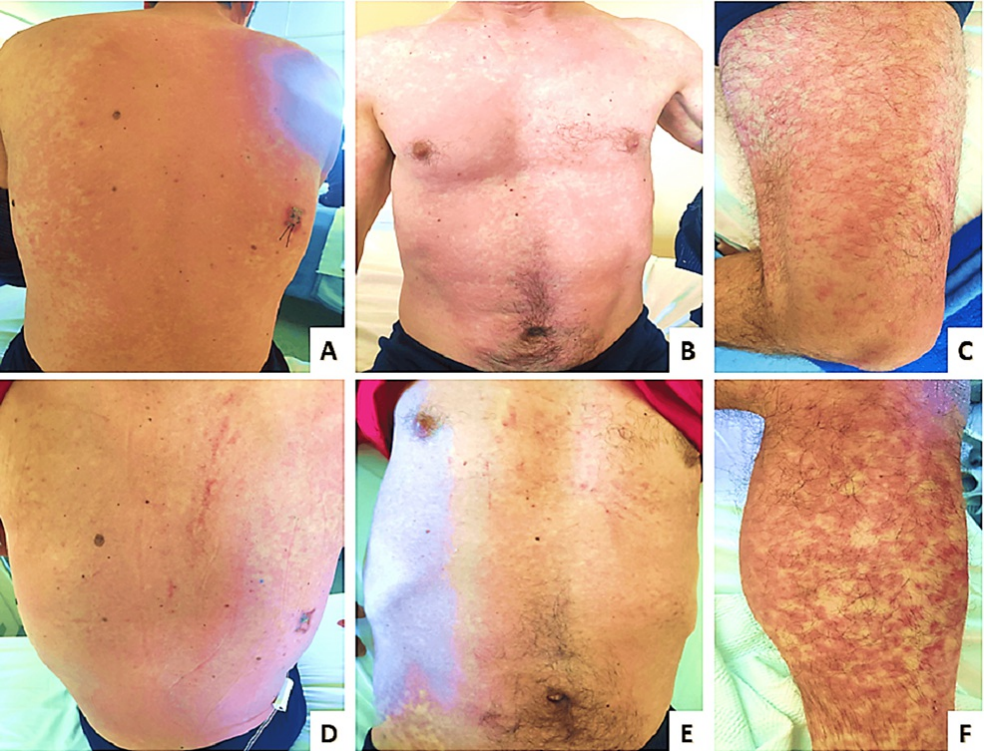
Inpatient evolving full body rash Maculopapular symmetrical rash on the trunk (A, B) and legs (C) turning to purpuric rash (D, E, F) the following day.

The patient developed a maculopapular symmetrical rash of the trunk and legs (Figure 4A, 4B, 4C) which morphed into a purpuric rash (Figure 4D, 4E, 4F) within 48 hours of fever onset covering more than 50% of the body surface area. Enlarged cervical and supraclavicular lymph nodes were palpable, acute phase reactants increased and a complete blood count comprised of eosinophilia (900 x 106 cells/mL), thrombocytopenia (120 x 103 platelets/μL) and atypical lymphocytes. Asymptomatic sinus rhythm bradycardia (38 beats per minute) without elevation of cardiac enzymes or pathological ultrasound findings was also present. Serology tests for chlamydia, mycoplasma, Epstein-Barr virus and cytomegalovirus, and autoimmune markers were all negative. Urine and blood cultures were negative for pathogens. A skin biopsy was obtained to rule out other diagnoses and after a multidisciplinary meeting, the diagnosis of drug reaction with eosinophilia and systemic symptoms (DRESS) syndrome was reached.

**Figure 5:**
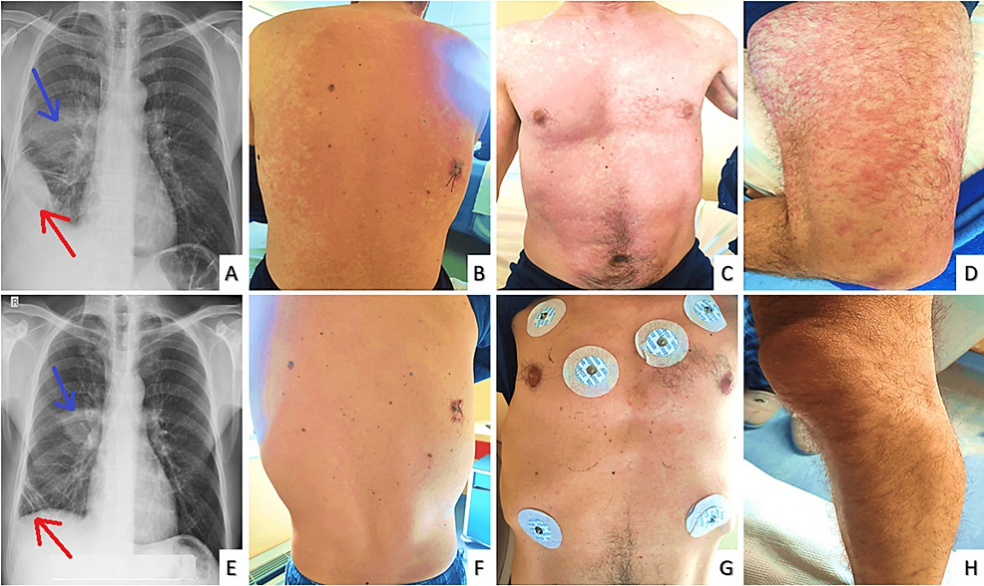
Chest x-rays on admission (A) & on discharge (E) and photos of the rash initially (B, C, D) and on discharge (F, G, H). Improved chest X-ray (red & blue arrows) on discharge (B) comparing to admission (A) and full remission of the rash on the trunk (B, C, F, G) and legs (D, H).

**Figure 6:**
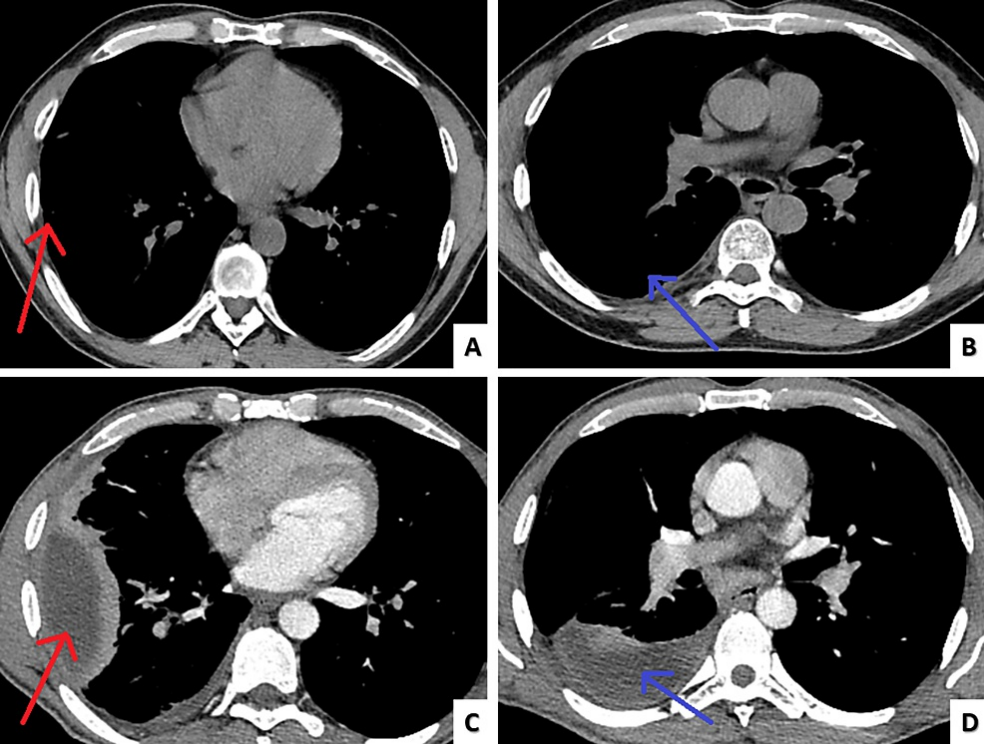
Outpatient follow up chest computed tomography one month after discharge Full remission of the two empyema pockets (red & blue arrow) (A, B) compared to the computed tomography on admission (red & blue arrow) (C, D).

After the diagnosis, piperacillin/tazobactam was immediately discontinued. Intravenous immunoglobulin and pulses of intravenous prednisolone were initiated due to concerns that sinus bradycardia could be a herald of eosinophilic myocarditis. After five days of intravenous prednisolone (125mg) pulses and as soon as the skin rash showed clinical improvement and sinus bradycardia reverted to sinus rhythm, intravenous doxycycline was carefully initiated as a substitute for b-lactam therapy. Corticosteroids were quickly tapered off after carefully considering the risk of progression of actinomycosis and DRESS relapse. The patient received a total of four weeks of intravenous antibiotic therapy under close in-hospital supervision and was then discharged and prescribed oral doxycycline (Figure 5). One month post-discharge, a repeat computed tomography demonstrated complete resolution of both effusions (Figure 6).

## Discussion

To the best of our knowledge, we hereby report the first case of actinomyces empyema complicated with DRESS syndrome. In this case of Actinomyces meyeri empyema, the initial non-invasive management with antibiotic therapy has been successfully followed because of five facts; i) effective early drainage through a chest tube; ii) no evidence of extensively disseminated disease; iii) absence of hemoptysis or bronchopleural fistulae; iv) no immediate need for decortication and v) adequate response to therapy. When the fever relapsed after three weeks of treatment, the first thought was Actinomycosis deterioration. Only after the improved CTs, the peripheral blood eosinophilia, and the rash appearance, the DRESS syndrome was suspected and then diagnosed.

Actinomycosis is a rare, chronic bacterial infection that induces suppurative, granulomatous inflammation. Involvement of the lung is evident in 15% of total cases and is thought to occur secondary to aspiration of oropharyngeal or gastrointestinal secretions [4]. Actinomyces israelii is the most common human pathogen, unlike Actinomyces meyeri whose isolation from patient samples has been described only in select case reports [5]. The propensity of the latter to infiltrate the respiratory system and then disseminate to other tissues is intriguing yet largely unexplained [6]. Advanced age, diabetes, alcohol abuse, chronic respiratory disorders, and poor dental hygiene are important factors that predispose individuals to the disease. A patient suffering from pulmonary actinomycosis typically presents with weight loss, productive cough, chest pain, and consolidation or mass-like lesion, which engender a broad differential including lung cancer and other chronic suppurative lung infections such as fungal infections, lung abscesses, and tuberculosis [7,8]. If left unchecked the infection may invade the pleura and result in actinomyces empyema, an extremely uncommon condition particularly when Actinomyces meyeri is the underlying cause.

Definitive diagnosis is elusive given the non-specific clinical/radiological findings and the challenging isolation of the bacteria from readily available clinical samples such as sputum and pleural fluid. The presence of sulfur granules in purulent material is a pathological hallmark of actinomycosis however they are also found in other conditions such as nocardiosis, coccidioidomycosis, and aspergillosis [9]. Upon high clinical suspicion, novel molecular methods can be employed; for instance, we amplified and sequenced microbial 16S ribosome RNA directly from the pleural fluid that showed 100% consistency with Actinomyces meyeri 16s rRNA.

Surgery is useful in controlling severe complications such as recurrent hemoptysis, bronchopleural fistulae, or restrictive lung disease following empyema. Invasive procedures may even be required when cancer cannot be ruled out or when antibiotic treatment alone fails to provide sufficient benefit. When promptly diagnosed, a significant number of patients respond favorably to treatment with high-dose intravenous penicillin for two to six weeks and then a prolonged regimen of penicillin V or amoxicillin for six to 12 months. The duration and administration route is decided on a case-by-case basis according to disease severity and clinical course [10,11]. More than 75% of cultures in which Actinomyces is isolated grow other organisms as well. As concomitant pulmonary infection of other anaerobic bacteria additionally to actinomycosis has been reported, treatment of the co-isolates is warranted depending on the organism. When known pathogens are isolated with Actinomyces, they can act synergistically, and in such cases, antimicrobial coverage for these additional organisms is mandatory, as copathogens may be recovered in culture. When broader therapy for coverage of copathogens is warranted, a combination beta-lactam plus beta-lactamase inhibitor is suggested such as piperacillin-tazobactam pr amoxicillin-clavulanate which offers the advantage of coverage against penicillin-resistant aerobic and anaerobic pathogens. Some copathogens can produce a beta-lactamase that can "shield" Actinomyces from penicillin and the addition of the beta-lactamase inhibitor is mandatory. Additional coverage may be warranted in cases of resistant pathogens. Therapy does not usually need to be directed against commensal flora which are cultured along with Actinomyces, since antimicrobial regimens effective against Actinomyces alone are usually curative unless foreign material is present [12].

Despite the well-documented safety of long-term b-lactam use in pulmonary actinomycosis patients, rare and even life-threatening side effects may develop. DRESS syndrome describes a hypersensitivity reaction to certain medications usually two to six weeks after exposure. The condition primarily involves rash, lymphadenopathy, fever, and characteristic hematologic abnormalities (eosinophilia and atypical lymphocytes). Elevated transaminases or hepatomegaly are also very common in light of the liver being the main site of internal organ involvement [13]. Although rare, cardiac insult bears significant mortality that mandates meticulous monitoring of patients with DRESS. Eosinophilic myocarditis should be suspected when elevation of cardiac biomarkers and/or changes related to electrocardiography or echocardiography are encountered. Treatment of DRESS relies on discontinuation of the offending agent and immunosuppression with systemic corticosteroids [14-16].

## Conclusions

We believe this work will help raise further awareness about rare infections such as actinomyces empyema complicated with severe allergic reactions such as DRESS syndrome and will be of particular interest to chest physicians as they are more likely to encounter such cases. High clinical suspicion is imperative in the setting of actinomycosis as timely identification favors effective resolution with long-term antibiotic therapy without the need for surgical intervention. During or after the antibiotic therapy, any findings of skin rash, eosinophilia, elevated transaminases, and relapse of fever should raise suspicion for drug reaction with eosinophilia and systemic symptoms syndrome. In that case, immediate discontinuation of the offending agent and systemic corticosteroids are the main options.
